# The Epiligament: Structure, Postnatal Development and Role in Ligament Healing

**DOI:** 10.7759/cureus.4836

**Published:** 2019-06-05

**Authors:** Nikola Stamenov, Paoleta Yordanova, Dimo Dimitrov, Militsa Telbiyska, Mark Stefanov

**Affiliations:** 1 Anatomy, Histology, and Embryology, Medical University of Sofia, Sofia, BGR; 2 Medicine, Medical University of Sofia, Sofia, BGR; 3 Anatomy, Medical University of Sofia, Sofia, BGR; 4 Anatomy, Medical University Sofia, Sofia, BGR

**Keywords:** epiligament, collagen, matrix metalloproteinases (mmps), ligament healing, review

## Abstract

While much is known about the ligament, the precise morphology and function of the thin layer of connective tissue lining its surface, termed the epiligament, have not been fully studied yet. Herein, we aimed at reviewing the recent findings on the structural and functional significance of the epiligament in both animal models and human tissue. The epiligament is made up of various connective tissue cells such as fibroblasts, fibrocytes, mast cells, and adipocytes and contains a number of neurovascular bundles. Arrangement of collagen fibers in the epiligament is rather chaotic, in multiple directions, which allows for greater mobility and resistance to stress. Differences in the collagen content and types of enzymes of the group of matrix metalloproteinases between the epiligament and the underlying ligament tissue have been reported and are reviewed herein. While the ligament tissue mainly contains collagen type I, the epiligament is also rich in collagen types III and V. As suggested by a number of studies, the epiligament plays a key role in ligament repair as a donor of cells and matrix metalloproteinases, particularly matrix metalloproteinase-2 and 9, which are essential for scar tissue remodeling. In conclusion, future studies will likely reveal additional functional aspects of the epiligament, which may allow scientists to devise more suitable treatment strategies for damaged ligaments in a world where injuries resulting from sports activities or daily routine have long merited their due attention.

## Introduction and background

Ligaments are solid bands made up of fascicles of collagen fibers which attach to the ends of two bones forming a joint and reinforce it. They are hypocellular and hypovascular structures that vary in size and shape [1]. While much is known about the ligament, the precise morphology and function of the thin layer of connective tissue lining its surface, termed the epiligament (EL), has remained clouded by obscurity and often neglected by researchers. Recent reports have shed more light on the histological and ultrastructural features of this structure and have suggested a number of significant roles which the EL plays in the processes of ligament nutrition, healing, and repair after injury [2-5]. Major differences were reported between the structural composition of the EL and the underlying ligament tissue (LT) [6-7]. In addition, the EL has been found to express various connective tissue components, as well as enzymes responsible for tissue breakdown and remodeling [8-11]. Herein, we aimed at reviewing the recent findings on the structural and functional significance of the EL both in animal models and in human tissue.

## Review

Histological and ultrastructural appearance of the EL

The EL was first described in the anterior cruciate (ACL), medial collateral (MCL), and lateral collateral ligament (LCL) of the rabbit knee by Bray et al. in 1990 as a ‘surrounding adherent connective tissue removed simultaneously with the ligament but which was grossly distinguishable from ligament tissue proper’ [12]. It is made up of various connective tissue cells - fibroblasts, fibrocytes, mast cells, and adipocytes and contains a number of neurovascular bundles [2,7,13-14]. In fact, most of the vessels in the EL-LT complex are located in the EL tissue. In addition, the EL contains an abundance of multidirectional collagen fibrils [14]. Two separate layers have been described in the EL, although they cannot be completely distinguished. One is composed of clustered fibroblasts, fibrocytes, fat cells, mast cells, nerves, and vessels. The second layer contains spindle-shaped and elongated fibroblasts, surrounded by collagen and a small number of blood vessels [4,13]. The EL extends towards the LT and forms the endoligament, which is a thin connective tissue layer enveloping the separate collagen fascicles in the LT [14].

EL fibroblasts have various shapes - small elongated ones, spindle-shaped, spinosus-shaped, and of irregular shape [13-14]. They contain large nuclei with delicate chromatin and prominent nucleoli. They are characterized by a high protoplasmic index and well-formed cytoplasmic outgrowths. The cytoplasm contains a significant number of ribosomes, polysomes, well-developed endoplasmic reticulum, poorly developed Golgi apparatus, single mitochondria and lysosomes [13-14]. Mast cells have prominent nuclei with peripheral heterochromatin. Their cytoplasm contains a number of typical round or oval electron-dense granules, enveloped by a membrane and separated from other granules through cytoplasmic septa [14]. Adipocytes, on the other hand, have an oval shape and appear ‘empty’ on routine histological examination due to the extraction of lipids as part of the tissue processing. Under the electron microscope, they were found to contain large lipid droplets. The rest of their cytoplasm is pushed to the periphery of the cell and their nuclei are eccentrically located [14].

Blood vessels in the EL are randomly dispersed, forming an extensively branching anastomotic network. They have thin walls and their intima consists of layers of endothelial cells surrounded by pericytes [15]. They are often accompanied by nerves, although they don’t always form neurovascular bundles [6,12,16-17]. Nerve fibers are both myelinated and unmyelinated and form nerve trunks, whose external fibrous coat (epineurium) fills the space between the bundles of nerve fibers, each surrounded by perineurium [15]. Schwann cells have also been described [15].

Similarities and differences between the EL and the LT

While the EL and LT constitute a single functional unit, differences in their structure are well manifested. As has been described above, the EL contains a multitude of connective tissue cells, blood vessels, and nerve fibers. In contrast, the ligament is poorly vascularized and made up of hypocellular sheets of longitudinal collagen fibers. While in the LT collagen fibers are oriented in parallel, in the EL their arrangement is rather chaotic, in multiple directions, which allows for greater mobility and resistance to stress. Both substructures are mainly built of collagen type I; however, the EL is also rich in collagen types III and V, which are usually only present in small amount in the intact LT [18-19].

Postnatal development of the EL

The first study of the postnatal development of the EL tissue was conducted by Georgiev and Vidinov in the LCL of the rat. In newborn animals, the EL consists of a small number of fibroblasts with typical shape, arranged either solitarily or in groups in the extracellular matrix [13]. The ultrastructure of the cells displays nuclei with delicate chromatin structure, developing rough endoplasmic reticulum and free ribosomes. The predominant type of collagen is type I. Blood vessels appear rather small and with thin walls. During the ongoing development of the animals, fibroblasts increase in number and chromatin in their nuclei becomes denser. The rough endoplasmic reticulum develops completely and large mitochondria appear. Collagen type I remains the predominant type and its fibers are grouped in bundles that are chaotically oriented. The wall of the blood vessels thickens and neurovascular bundles are formed with adjacent nerve elements. As the cells and neurovascular bundles increase in number, two layers are formed. The first one consists of fibroblasts, adipocytes, myelinated and unmyelinated nerve fibers, and blood vessels; the second layer consists of spindle-shaped and elongated fibroblasts, as well as solitary blood vessels.

Role of the EL in ligament healing

It has been shown that as a more cellular structure the EL may play a central role in the formation of scar tissue and the healing process of the ligament [16]. Fibroblasts in the EL are not static cells; instead, they produce varying quantities of collagen types I, III, and V, fibronectin, and enzymes of the group of matrix metalloproteinases (MMPs), especially MMP-2 and 9 [8-10,19-21]. These substances are all upregulated to promote adequate ligament repair after injury. In addition, injuries can stimulate the release of other cell types such as neutrophils, macrophages, T-lymphocytes and hematopoietic cells, along with various growth factors [22]. In different parts of the body, these cells have a major role in the process of healing, which shows that they can be involved in the differentiation, phagocytosis, and synthesis of collagen in the healing ligament, too [22].

The recovery of the injured tissue passes through several stages. Initially, the zone of the injury represents an extremely disorganized tissue, with intense angiogenesis and marked hypercellularity, the main cells being fibroblasts and progenitor cells [23]. They have a basophilic nucleus, with a slightly colored vitreous cytoplasm. A study of their ultrastructure reveals an increase in the number of lysosomes - a sign of active phagocytosis, as well as a multitude of spherical mitochondria - a sign of active metabolism [7]. Most cells of the deep layer of the EL extend to the endoligament. As the tissue develops, hypercellularity gradually decreases. Fibroblasts and progenitor cells migrate into the EL, becoming better differentiated, adipocytes are of irregular size, and blood vessels are beginning to decrease [20]. Eventually, on day 30 after injury, the process of recovery of the damaged tissue is complete [20]. The process of ligament healing after the injury is marked by the formation of scar tissue rather than regeneration, which is a common mechanism to other soft tissues [1,24-26].

A recent paper by Georgiev et al. compared the spontaneous healing of the rat MCL after injury with healing through ligament suture application [27]. Histological and ultrastructural changes were observed in the EL on the 8th, 16th and 30th post-injury day and showed consistency with previous reports. Initially, the EL was characterized by hypercellularity, with the predominance of active collagen-synthesizing fibroblasts, whose numbers returned to normal values by day 30. The authors reported no statistically significant difference in the number of cells between rats healing spontaneously and those healing after suture application, which supported the hypothesis of the role of the EL in MCL repair and provided another explanation for the good potential of the MCL for spontaneous healing after injury.

Types of collagen expressed in the EL

Approximately 75% of the dry weight of ligaments is represented by collagen [28]. Collagen type I is the major constituent (nearly 85%) and is chiefly responsible for the tensile strength of the ligament and the long-term properties of the matrix [29-30]. Other collagens include types III, V, VI, XI, and XIV [28]. Unlike type I, collagen type III is more flexible and is responsible for the higher extensibility of the ligament [31]. It is implicated in ligament repair and its synthesis is gradually increased after ligament injury up to the point where its levels surpass those of collagen type I in the areas of rapid healing [18,28,32-34]. Collagen type V has a role in cell migration and growth kinetics and is also upregulated during ligament recovery [24,35]. It plays a major role as a regulator of collagen fibril diameter and in the remodeling of the extracellular matrix. Collagen type XIV is responsible for linear fibril growth [34]. As indicated above, a major difference between the EL and the LT is that the former is rich in types III and V, which are only expressed in high quantities in the LT after damage to the ligament [18-19]. Furthermore, there appears to be a difference in the collagen content of the EL of different ligaments. Georgiev et al. [19] conducted a comparative semi-quantitative analysis of the expression of collagen types I and V and procollagen type III in the EL of the MCL and ACL and noted a stronger immunohistochemical reaction in the EL of the MCL for all three studied molecules. The authors discussed that this could be a possible reason for the better healing capacity of the MCL compared to the ACL. In addition, they confirmed the earlier reports of stronger expression of types III and V in the EL than in the LT.

MMPs expressed in the EL

MMPs are a group of calcium- and zinc-dependent endopeptidases, which play a key role in the growth, morphogenesis, and remodeling of connective tissue by breaking down and removing extracellular matrix molecules, splitting and remodeling the molecules of collagen, elastin, casein and gelatin [36,37]. Thus, MMPs are implicated in a number of physiological and pathological processes throughout the body, including wound healing, ligament repair, myocardial remodeling induced by hypertension, tumor invasion, and metastasis and many others [36-40]. They are secreted by various cells - fibroblasts, osteoblasts, endothelial cells, macrophages, neutrophils, lymphocytes [10]. Karousou et al. were among the first to study the expression of MMPs in the ligament and found higher levels of MMP-2 and 9 in the zone of injury in human Achilles tendon as opposed to a healthy zone [41]. Later, Zhang et al. compared MMP expression between the MCL and ACL and found that the levels of MMP-1, 2, 14, 17, and 23 are significantly higher in the MCL, while MMP-3 is higher in the ACL [42]. They concluded that the differential expression of MMPs between the two ligaments is tied to their healing potential. However, these studies did not distinguish between MMP expression in the EL and the LT. Iliev et al. reported MMP-2 and 9 expression in the EL of the MCL in healthy rats and found that it was located in the fibroblasts in both substructures, as well as in the adventitia of blood vessels and the perivascular zone of the EL, with MMP-2 expression being stronger [8]. Georgiev et al. studied MMP-2 expression in the MCL after injury and reported that the immunoreactivity of the enzyme remained strong even on the 30th post-injury day and was localized predominantly in the EL [10]. Similar findings were reported for MMP-9 [9]. These findings are inconclusive; however, they show the role of the MMPs expressed in the EL in the regeneration of the ligament and support their significance for the healing potential of individual ligaments.

## Conclusions

In recent years, interest in the EL, the superficial connective tissue layer covering the LT proper, has peaked. Reports in the literature have proved its role as a donor of cells and enzymes responsible for the proper functioning of the ligament and its repair after injury. Its histological and ultrastructural features, along with its collagen content have been well characterized both in experimental animal models and in human tissue. It appears that the EL plays a pivotal role in the healing potential of individual ligaments. Despite these discoveries, future studies will likely reveal additional functional aspects of the EL, which may allow scientists to devise more suitable treatment strategies for damaged ligaments in a world where injuries resulting from the sport activities or daily routine have long merited their due attention.
